# Oriented Neural Spheroid Formation and Differentiation of Neural Stem Cells Guided by Anisotropic Inverse Opals

**DOI:** 10.3389/fbioe.2020.00848

**Published:** 2020-07-31

**Authors:** Lin Xia, Yixuan Shang, Xiangbo Chen, He Li, Xiaochen Xu, Wei Liu, Guang Yang, Tian Wang, Xia Gao, Renjie Chai

**Affiliations:** ^1^MOE Key Laboratory for Developmental Genes and Human Disease, Institute of Life Sciences, Jiangsu Province High-Tech Key Laboratory for Bio-Medical Research, Southeast University, Nanjing, China; ^2^Department of Clinical Medical Engineering, Nanjing Drum Tower Hospital, The Affiliated Hospital of Nanjing University Medical School, Nanjing, China; ^3^Key Laboratory of Molecular Epigenetics of the Ministry of Education, Northeast Normal University, Changchun, China; ^4^Hangzhou Rongze Biotechnology Group Co., Ltd., Hangzhou, China; ^5^Department of Otorhinolaryngology Head and Neck Surgery, The First Affiliated Hospital of Wenzhou Medical University, Wenzhou, China; ^6^Department of Otolaryngology-Head and Neck Surgery, The Second Xiangya Hospital, Central South University, Changsha, China; ^7^Department of Otorhinolaryngology, Affiliated Sixth People’s Hospital of Shanghai Jiao Tong University, Shanghai, China; ^8^Department of Otolaryngology-Head and Neck Surgery, Nanjing Drum Tower Hospital, The Affiliated Hospital of Nanjing University Medical School, Jiangsu Provincial Key Medical Discipline (Laboratory), Research Institute of Otolaryngology, Nanjing, China; ^9^Co-Innovation Center of Neuroregeneration, Nantong University, Nantong, China; ^10^Institute for Stem Cell and Regeneration, Chinese Academy of Sciences, Beijing, China

**Keywords:** neural regeneration, neural stem cells, neurons, oriented growth, anisotropic inverse opal substrate

## Abstract

Isotropic inverse opal structures have been extensively studied for the ability to manipulate cell behaviors such as attachment, migration, and spheroid formation. However, their use in regulate the behaviors of neural stem cells has not been fully explored, besides, the isotropic inverse opal structures usually lack the ability to induce the oriented cell growth which is fundamental in neural regeneration based on neural stem cell therapy. In this paper, the anisotropic inverse opal substrates were obtained by mechanically stretching the poly (vinylidene fluoride) (PVDF) inverse opal films. The anisotropic inverse opal substrates possessed good biocompatibility, optical properties and anisotropy, provided well guidance for the formation of neural spheroids, the alignment of neural stem cells, the differentiation of neural stem cells, the oriented growth of derived neurons and the dendritic complexity of the newborn neurons. Thus, we conclude that the anisotropic inverse opal substrates possess great potential in neural regeneration applications.

## Introduction

Neurodegenerative disorders are usually caused by loss or dysfunction of neurons that results in the disruption of signal transduction in the nervous system such as neurosensory deafness, Parkinson’s disease, and Huntington’s disease (Lindvall and Kokaia, 2010). Repairing the neural circuit by replacement of the dysfunctional neurons would represent an effective cure for neurodegenerative disorders. Nowadays, based on stem cell transplantation, neural stem cells which possess self-renewal and pluripotency have become a promising curative agent for neurodegenerative disorders treatment (Lindvall et al., 2004; McGinley et al., 2018; Reidling et al., 2018; Song et al., 2018; Yang et al., 2018; Ahmadian-Moghadam et al., 2020; Derakhshankhah et al., 2020; Xapelli et al., 2020). Following transplantation, neural stem cells can differentiate into neural subtype cells, including neurons, astrocytes, and oligodendrocytes, *in vivo* (Gage, 2000). However, because neural circuits have specific orientations (Rose et al., 2017), in order to repair the neural circuits and recover the transduction of neural signals, the newborn neurons need not only normal functionality, but also must grow towards the target cells to form synaptic connections. Thus the guided growth of newborn neurons has become the critical factor for the use of neural stem cell transplantation in neural regeneration.

With the development of biomaterials, topography has become a promising physical cue for manipulating cell behaviors during neural regeneration (Guo et al., 2016; He et al., 2016; Jiang et al., 2016; Severino et al., 2016; Liu et al., 2018; Han et al., 2019; Tang et al., 2019; Xia et al., 2019; Fang et al., 2020). The topography of these materials influences the mechanosensory apparatus and the spatiotemporal dynamics of the cells (Chen et al., 2014), and these cell-material interactions play a key factor in cell behavior regulation (Guilak et al., 2009; Zangi et al., 2016). Many kinds of biomaterials have been investigated for guiding cell growth through topography, including nanofibers (Liu et al., 2010; Xie et al., 2014; Omidinia-Anarkoli et al., 2017; Zuidema et al., 2018; Li et al., 2019), colloidal nanoparticles (Antman-Passig et al., 2017; Musoke-Zawedde and Shoichet, 2006), and inverse opal materials (Lu et al., 2014; Shang et al., 2019; Li et al., 2020). Among the applied biomaterials, inverse opal materials represent a class of porous structures with an ordered array of uniform nanoscale or microscale pores, which possessed well-controlled pore size, long-range ordered structure, and homogeneous interconnectivity. On the other side, the 3D porous structure of the inverse opal materials is very facilitated to the distribution of oxygen/nutrients/cells (Zhang and Xia, 2012). Thus, the inverse opal materials have been widely investigated in biomedical applications such as cellular co-culture (Kim et al., 2014; Im et al., 2017; Mushtaq et al., 2019), cell migration (Stachowiak and Irvine, 2008; Zhang et al., 2013; Mushtaq et al., 2019), and fabrication of multicellular spheroids (Zhang and Xia, 2012; Zhang et al., 2017). However, their application in guiding the oriented growth of neurons has not been fully explored.

In this study, we designed the anisotropic inverse opal substrates with elliptical macro-pores using mechanical stretching. The substrates were fabricated with PVDF, which possesses well piezoelectricity and has been widely applied in biomedical and flexible electronic devices. The neural stem cell spheroids cultured on the anisotropic inverse opal substrates exhibited good proliferation, and the cultured neural stem cells were induced into an ordered alignment and the newborn neurons showed oriented growth. In addition, the dendritic complexity index (DCI) of the newborn neurons was also significantly increased under the oriented guidance of the anisotropic inverse opal substrates. These features indicate the wide biomedical applications of the anisotropic inverse opal substrates.

## Results and Discussion

### Materials Characterization

**FIGURE 1:**
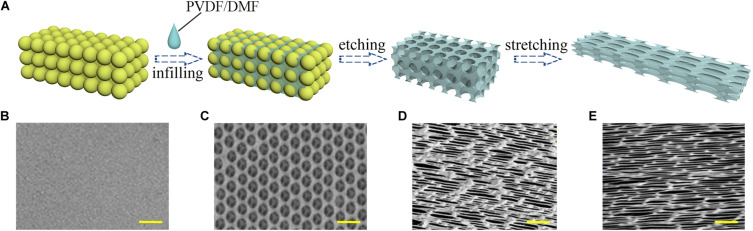
Materials characterization. **(A)** Schematic illustration of the manufacturing process of the anisotropic inverse opal substrates; the SEM images of the PVDF control substrate **(B)**, the inverse opal substrate **(C)**, the 3× stretched substrate **(D)**, and the 6× stretched substrate **(E)**. Scale bars are 1 mm in panels **(B–E)**.

The fabrication of the inverse opal substrate was based on a colloidal silica crystal template. As shown in Figure 1A, the template was manufactured by the vertical deposition of silica nanoparticles on a glass following by sintering under 500°C. A solution of PVDF material dissolved in dimethylformamide (DMF) was used to fill the void space of the template. The PVDF solidified after the evaporation of the DMF, and the silica nanoparticles were dissolved by hydrofluoric acid. Thus a PVDF inverse opal substrate with highly ordered pore array was obtained (Figure 1C). To generate anisotropy, the PVDF inverse opal substrate underwent mechanical stretching along the uniaxial orientation. As shown in Figures 1D,E, the lengths increased 3× and 6× under stretching, and the pores of the inverse opal materials became ellipses. A flat PVDF film without any topographical features was fabricated as the control substrate (Figure 1B). This PVDF film possessed the same material with the inverse opal substrates, thus to exclude the influence of fabricating material in comparison to the inverse opal substrates.

### Optical Properties of the Substrates

Due to the periodicity of the macropores, the inverse opal substrates could regulate the photon propagation by the photonic band gap (PBG), thus exhibiting the structures with different colors. This coloration derived from the interference between visible light and structural features is called “structural color.” The periodic elliptical structures in the stretched anisotropic inverse opal substrates also possessed photon-manipulating capability. Thus, the light with certain wavelengths could be blocked and reflected by the PBG. The reflecting peak of the structural colors could be described by the Bragg–Snell equation:

λ=1.633D(n-2averagecosθ2)21/

**FIGURE 2:**
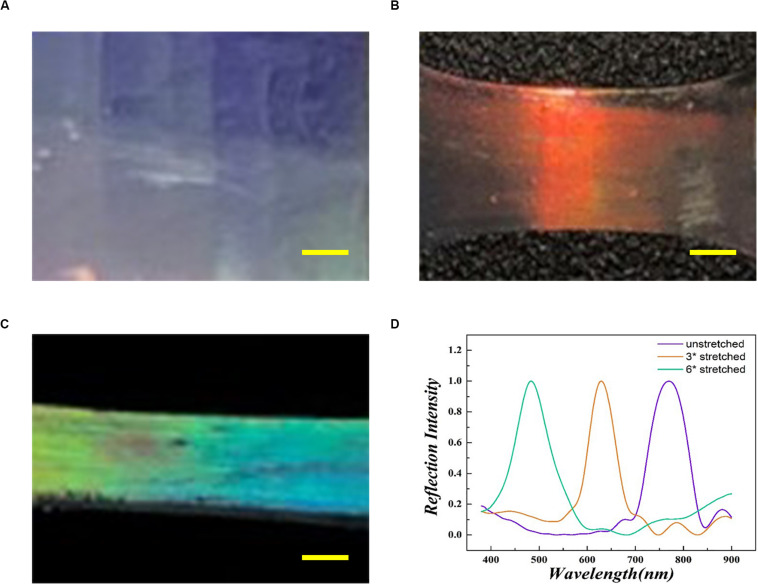
Optical images of the inverse opal substrate **(A)**, the 3× stretched substrate **(B)**, and the 6× stretched substrate **(C)**. **(D)** The reflecting peaks of the inverse opal substrates. The purple line matches with panel **(A)**, the orange line matches with panel **(B)**, and the green line matches with panel **(C)**. Scale bars are 1 mm in panels **(A–C)**.

In the equation, D represents the distance with the diffracting point, *n*_average_ represents the average index of refraction, and θ represents the incident Bragg glancing angle. According to the equation, changes in D or θ are effective approaches to regulate the structural color. As shown in Figure 2, as the degree of stretching increased, the round pores of the substrates gradually became ellipses leading to decreased values of D along the stretch orientation. This led to a blue-shift in the reflective peak with a fixed θ (Figure 2D). The blue-shift indicated the increase of the wavelength, which meant that the wavelength of reflective light was increased by the PBG of substrates. This excellent optical property indicates the huge potential of these substrates in biomedical applications such as detection, sensors and decoration (Zhu et al., 2017). However, their application in neural regeneration has not been fully explored.

### Promoted Formation of Neural Stem Cell Spheroids

**FIGURE 3:**
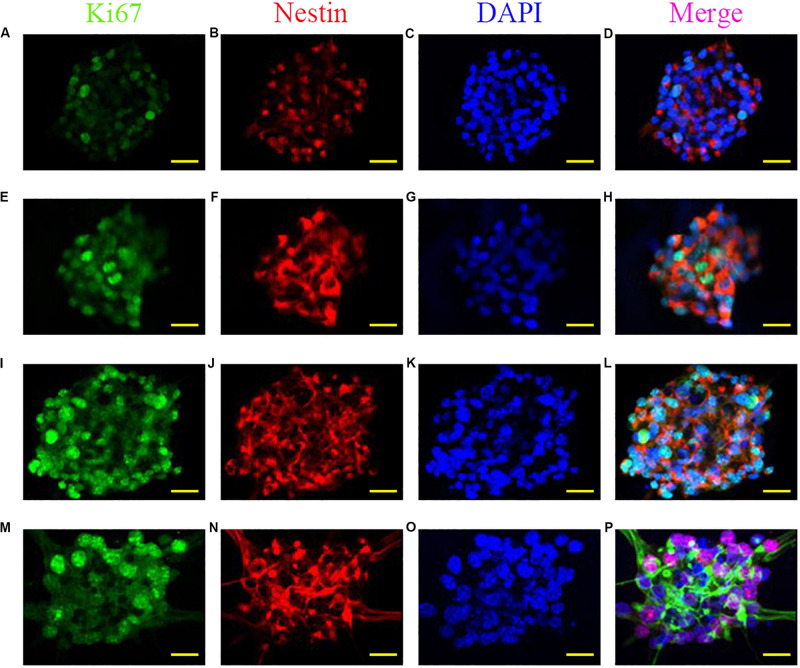
Promoted formation of neural stem cell spheroids. Fluorescent images of Ki67, Nestin, DAPI, and merged images of neural stem cell spheroids cultured on the PVDF control **(A–D)**, on the inverse opal substrate **(E–H)**, on the 3× stretched substrate **(I–L)**, and on the 6× stretched substrate **(M–P)**. Scale bars are 20 μm.

The ability of self-renewal is a critical factor for neural stem cells applied in neural regeneration. To explore the influence of the anisotropic inverse opal substrates on self-renewal ability, neural stem cell spheroids were seeded onto the anisotropic inverse opal substrates. A flat PVDF film without any topographical structures was manufactured as the control. In these experiments the complexity of serum components makes it difficult to maintain the undifferentiated state of neural stem cells in serum-containing medium, so the seeded neural stem cell spheroids were cultured under proliferation conditions in serum-free medium. After 7 days of culture, Ki67 immunofluorescence staining was used to measure the proliferation of neural stem cell spheroids, and Nestin immunofluorescence staining was used to measure the pluoripotency of the neural stem cells. As shown in Figure 3, the diameters of neural stem cell spheroids increased with the degree of stretching of the substrates, the number of Ki67 cells significantly increased with the degree of stretching, and Nestin staining indicated that the neural stem cells maintained pluoripotency during the culture. In addition, the statistics of the spheroid diameters and the Ki67-positive cells were shown in Supplementary Figure S1, which indicates that the inverse opal substrates promoted the neural stem cell spheroids formation.

### Alignment of Neural Stem Cells

**FIGURE 4:**
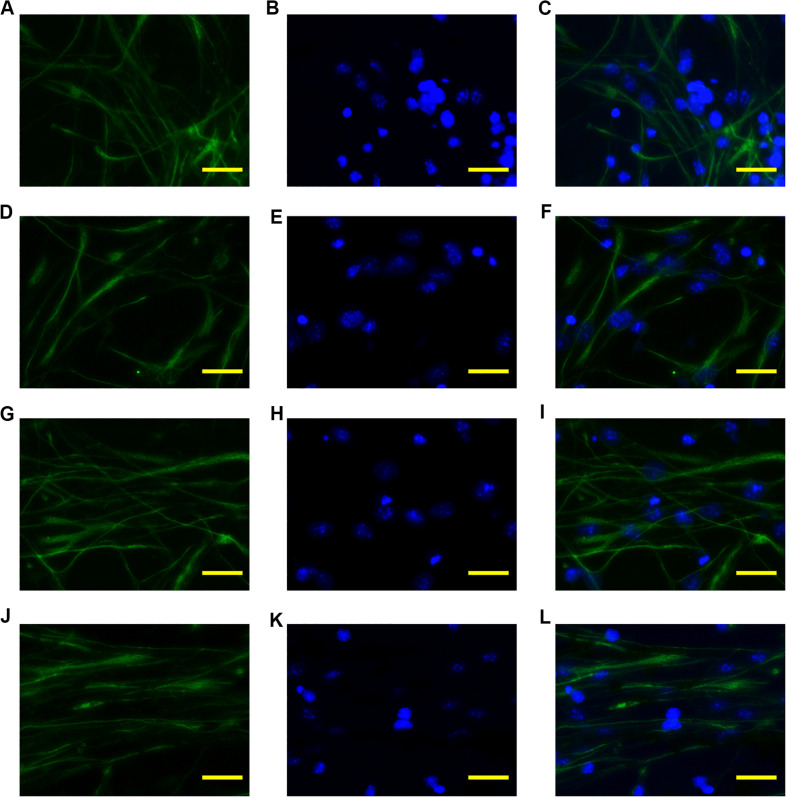
Alignment of neural stem cells. The fluorescent images of Nestin, DAPI, and merged images of the neural stem cells cultured on the PVDF control **(A–C)**, on the inverse opal substrate **(D–F)**, on the 3× stretched substrate **(G–I)**, and on the 6× stretched substrate **(J–L)**. Scale bars are 20 μm.

To investigate the guiding effect of the anisotropic inverse opal substrates, monodispersed neural stem cells were seeded onto the surfaces of the inverse opal substrates and cultured under proliferation conditions. The neural stem cells adhered to the substrates and migrated according to the topographical induction of the substrates. After 5 days of culture, Nestin immunofluorescence staining was used to visualize the seeded neural stem cells and assess their viability at the same time. As shown in Figure 4, the neural stem cells were random on the PVDF control, which was similar with the neural stem cells seeded on the inverse opal substrate without stretching. However, with increasing degree of stretch, the orientations of the neural stem cells showed greater alignment on the anisotropic inverse opal substrates. The statistic of orientation angles of the neural stem cells validated the fluorescent images, 51.4 and 71.6% of the seeded neural stem cells showed orientation angles within 10° of the stretch direction in the 3× and 6× stretched anisotropic inverse opal substrates, respectively (Supplementary Figure S1b). This illustrated the strong guiding effect of the anisotropic inverse opal substrates.

### Differentiation of Neural Stem Cells

**FIGURE 5:**
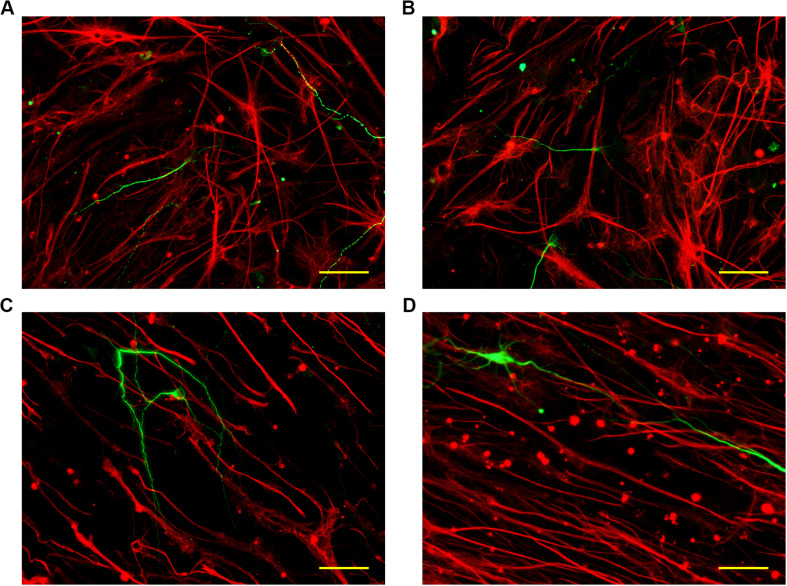
Differentiation of neural stem cells. The fluorescent images of neurons and glial cells cultured on the PVDF control **(A)**, on the inverse opal substrate **(B)**, on the 3× stretched substrate **(C)**, and on the 6× stretched substrate **(D)**. Scale bars are 40 μm.

To explore the differentiation of neural stem cells under the guidance of topographical cues, monodispersed neural stem cells were seeded onto the inverse opal substrates. After culture for 24 h under proliferation conditions, the neural stem cells adhered to the surface of the inverse opal substrates. The culture was then changed to differentiation conditions with serum-containing medium. After culturing for 7 days, Tuj1 and GFAP immunofluorescence staining was used to visualize the newborn neurons (green) and the glial cells (red). As shown in Figure 5, the orientations of the newborn neurons and the glial cells were random in the control substrate and the inverse opal substrate without stretching. However, with increasing degree of stretching, the orientations of differentiated cells showed alignment on the inverse opal substrate. In statistics, 49 and 68% of the newborn neurons showed orientation angles within 10° of the stretch direction on the inverse opal substrates with 3× and 6× stretching, respectively (Supplementary Figure S2b). This effect showed the strong guiding effect of the anisotropic topographical cues of the inverse opal substrates on the oriented growth of newborn neurons. In addition, the percentage of newborn neurons was also increased under the guidance of the anisotropic inverse opal substrates (Supplementary Figure S2a). Furthermore, the morphological properties of the newborn neurons were assessed as shown in Supplementary Figure S3. The morphological properties of the newborn neurons on the inverse opal substrate without stretching were similar to those cultured on the PVDF control. However, as the degree of stretching increased, the average branch length increased, while the average number of primary dendrites and the average number of branch points decreased. This effect was induced by the “contact-guidance,” the growth cones of the neurites measured the guiding cues of the inverse opal substrates, which led the oriented extension of the neurites, thus stimulate the increase of branch length. At the same time, the oriented extension of the branch decreased the average number of primary dendrites and the average number of branch points. These changes increased the DCI of the newborn neurons, which indicated that the dendritic complexity of the newborn neurons was increased under the guidance of the topographical cues of the anisotropic inverse opal substrates.

## Conclusion

The topological cues provided by biomaterials have become a promising approach to regulate the fate of neural stem cells. Inverse opal substrates possess periodic pore structures and have excellent ability to regulate cell behaviors. However, these substrates have rarely been used to regulate the fate of neural stem cells, which is important for neural regeneration. In this study, inspired by the stretching property of the PVDF material, we investigated how changes in the topographical cues of the inverse opal substrate due to stretching the material might regulate the fate of neural stem cells. We show that the anisotropic inverse opal substrates possess good biocompatibility and can maintain the pluoripotency of the seeded neural stem cells during culture. In addition, in comparison to the isotropic inverse opal substrate, the anisotropic inverse opal substrates showed greater proliferation of the neural stem cell spheroids, oriented alignment of the monodispersed neural stem cells, ordered alignment of differentiated neural cells, and increased DCI of newborn neurons. These features indicate the huge potential of the anisotropic inverse opal substrates for guiding the arrangement of differentiated cells for neural stem cell-based neural regeneration.

Furthermore, due to the soft polymer PVDF material, the anisotropic inverse opal substrates can deform as cells grow on them, and this leads to shifts in the reflection peak of the material. This self-reporting property of the anisotropic inverse opal substrates suggests that these materials might make excellent detectors when integrated into various platforms such as microfluidics. Together, these results indicated the well biomedical applied prospect of anisotropic inverse opal substrates.

## Materials and Methods

### Materials

Monodispersed silicon dioxide spheres were purchased from the company of Nanorainbow Biotechnology (Jiangsu China), Polyvinylidene fluoride (PVDF) and Laminin were purchased from the company of Sigma Aldrich (MO, United States). N, N-dimethylformamide (DMF) was purchased from the company of Sinopharm Chemical Reagent (Shanghai, China). Ethanol and hydrofluoric acid were purchased from the company of Aladdin (Shanghai, China). Penicillin-streptomycin, Accutase, B-27 supplement (50×), EGF (recombinant human epidermal growth factor), DMEM/F-12(1:1) medium, PBS (Phosphatebuffered saline), and HBSS (Hank’s balanced salt solution) were purchased from the company of Gibco (NY, United States). FGF (recombinant murine FGF-basic) was purchased from the company of PeproTech (NJ, United States). The NeuroCult^TM^ Differentiation Kit (Mouse) was purchased from the company of Stemcell (CA, United States).

### Neural Stem Cell Isolation, Proliferation, and Differentiation

#### Neural Stem Cell Isolation

Neural stem cells were isolated from hippocampuses obtained from 16 to 18-day embryonic rats, and the isolated hippocampuses were collected in a petri dish of HBSS under 4°C and then rinsed twice with HBSS. The isolated hippocampuses were digested with Accutase for 20 min under 37°C then triturated gently with a pipette tip. The monodispersed neural stem cells were suspended in medium (2% B-27 dissovled in DMEM/F12), then cultured under 37°C with 5% CO_2_.

#### Neural Stem Cell Proliferation

The neural stem cells were seeded in proliferation medium (2% B-27, 20 ng/ml EGF, 20 ng/ml FGF dissovled in DMEM/F12) at 5 × 10^4^ cells/ml. The neural stem cells were then cultured in a humidified atmosphere under 37°C with 5% CO_2_ and passaged every 7 days.

#### Neural Stem Cell Differentiation

The neural stem cells were cultured in differentiation medium (preparation from the NeuroCult^TM^ Differentiation Kit (Mouse) according to the recommendation) at 5 × 10^4^ cells/ml. The differentiation medium was replaced every 2 days after the neural stem cells had firmly attached onto the inverse opal substrates.

#### Immunofluorescence Staining

The cells were washed with PBS three times then fixed in 4% paraformaldehyde for 30 min, blocked with blocking buffer for 1 h under room temperature, stained with primary antibodies against Nestin, Tuj1, or GFAP overnight under 4°C, and stained with FITC or rhodamine-conjugated secondary antibodies for 1 h under room temperature. The nuclei were stained with DAPI for 15 min under room temperature.

#### Preparation of the Substrates

Monodispersed silica dioxide spheres were vertically deposited on the glass coverslip and sintered under 500°C for 4 h to fabricate the silica colloidal crystal templates. The solution of PVDF/DMF (0.04 g/ml) was infiltrated into the templates. After the evaporation of DMF, the PVDF became solidified. Then, the silica nanoparticles were etched by hydrofluoric acid (2% wt), thus forming the inverse opal PVDF films. The PVDF films were uniaxially stretched using Vernier caliper (Masterproof) to 3× and 6× elongation in an 80°C water bath. The stretched PVDF substrates were observed by scanning electron microscopy (Hitachi S-300N) and with a fluorescence microscope (Leica DM5000). On the contrary, the solution of PVDF/DMF (0.04 g/ml) was added onto the glass and evaporated under room temperature, after the evaporation of DMF, the PVDF became solidified, thus forming the flat PVDF film without pores as the control substrate. All the substrates were coated by Laminin (1%) before the seeding of neural stem cells.

#### Statistical Analysis

The data was analyzed by SPSS software, the results were achieved by three independent parallel experiments, the significant was considered at *P* < 0.05.

## Data Availability Statement

All datasets presented in this study are included in the article/Supplementary Material.

## Author Contributions

LX, GY, TW, XG, and RC were responsible for the study design and implementation, data analysis, and manuscript writing. LX, YS, XC, HL, and XX were responsible for the data collection. LX, YS, XC, HL, and XX were responsible for the statistical analysis. WL played an important role in study design and experiment. All authors contributed to the article and approved the submitted version.

## Conflict of Interest

XC was employed by company Hangzhou Rongze Biotechnology Group Co., Ltd.

The remaining authors declare that the research was conducted in the absence of any commercial or financial relationships that could be construed as a potential conflict of interest.

## References

[B1] Ahmadian-Moghadam H. Sadat-Shirazi M. S. Zarrindast M. R. (2020). Therapeutic potential of stem cells for treatment of neurodegenerative diseases. *Biotechnol. Lett.* 10.1007/s10529-020-02886-1 32342435

[B2] Antman-Passig M. Levy S. Gartenberg C. Schori H. Shefi O. (2017). Mechanically oriented 3D collagen hydrogel for directing neurite growth. *Tissue Eng. Part A.* 23 403–414. 10.1089/ten.tea.2016.0185 28437179

[B3] Chen W. Shao Y. Li X. Zhao G. Fu J. (2014). Nanotopographical surfaces for stem cell fate control: engineering mechanobiology from the bottom. *Nano Today.* 9 759–784. 10.1016/j.nantod.2014.12.002 25883674 PMC4394389

[B4] Derakhshankhah H. Sajadimajd S. Jafari S. Izadi Z. Sarvari S. Sharifi M. (2020). Novel therapeutic strategies for Alzheimer’s disease: implications from cell-based therapy and nanotherapy. *Nanomed. Nanotechnol. Biol. Med.* 24:102149. 10.1016/j.nano.2020.102149 31927133

[B5] Fang Q. J. Zhang Y. H. Chen X. B. Li H. Cheng L. Y. Zhu W. J. (2020). Three-dimensional graphene enhances neural stem cell proliferation through metabolic regulation. *Front. Bioeng. Biotechnol.* 7:436. 10.3389/fbioe.2019.00436 31998703 PMC6961593

[B6] Gage F. H. (2000). Mammalian neural stem cells. *Science* 287 1433–1438. 10.1126/science.287.5457.1433 10688783

[B7] Guilak F. Cohen D. M. Estes B. T. Gimble J. M. Liedtke W. Chen C. S. (2009). Control of stem cell fate by physical interactions with the extracellular matrix. *Cell Stem Cell* 5 17–26. 10.1016/j.stem.2009.06.016 19570510 PMC2768283

[B8] Guo R. Zhang S. Xiao M. Qian F. He Z. Li D. (2016). Accelerating bioelectric functional development of neural stern cells by graphene coupling: implications for neural interfacing with conductive materials. *Biomaterials* 106 193–204. 10.1016/j.biomaterials.2016.08.019 27566868

[B9] Han S. Sun J. He S. B. Tang M. L. Chai R. J. (2019). The application of graphene-based biomaterials in biomedicine. *Am. J. Transl. Res.* 11 3246–3260. 31312342 PMC6614642

[B10] He Z. H. Zhang S. S. Song Q. Li W. Y. Liu D. Li H. W. (2016). The structural development of primary cultured hippocampal neurons on a graphene substrate. *Colloids Surfaces B Biointerfaces* 146 442–451. 10.1016/j.colsurfb.2016.06.045 27395037

[B11] Im P. Ji D. H. Kim M. K. Kim J. (2017). Fabrication of cell-benign inverse opal hydrogels for three-dimensional cell culture. *J. Colloid Interface Sci.* 494 389–396. 10.1016/j.jcis.2017.01.108 28171847

[B12] Jiang Z. Y. Song Q. Tang M. L. Yang L. Y. Cheng Y. L. Zhang M. (2016). Enhanced migration of neural stem cells by microglia grown on a three-dimensional graphene scaffold. *ACS App. Mater. Interfaces* 8 25069–25077. 10.1021/acsami.6b06780 27589088

[B13] Kim J. Bencherif S. A. Li W. A. Mooney D. J. (2014). Cell-friendly inverse opal-like hydrogels for a spatially separated co-culture system. *Macromol. Rapid. Commun.* 35 1578–1586. 10.1002/marc.201400278 25113941 PMC4318565

[B14] Li G. F. Chen K. You D. Xia M. Y. Li W. Fan S. N. (2019). Laminin-coated electrospun regenerated silk fibroin mats promote neural progenitor cell proliferation. Differentiation, and survival in vitro. *Front. Bioeng. Biotechnol.* 7:190. 10.3389/fbioe.2019.00190 31448271 PMC6691020

[B15] Li L. J. Chen Z. Y. Shao C. M. Sun L. Y. Sun L. Y. Zhao Y. J. (2020). Graphene hybrid anisotropic structural color film for cardiomyocytes’ monitoring. *Adv. Funct. Mater.* 30:1906353 10.1002/adfm.201906353

[B16] Lindvall O. Kokaia Z. (2010). Stem cells in human neurodegenerative disorders - time for clinical translation? *J. Clin. Invest.* 120 29–40. 10.1172/jci40543 20051634 PMC2798697

[B17] Lindvall O. Kokaia Z. Martinez-Serrano A. (2004). Stem cell therapy for human neurodegenerative disorders - how to make it work. *Nat. Med.* 10 S42–S50. 15272269 10.1038/nm1064

[B18] Liu X. A. Chen J. Gilmore K. J. Higgins M. J. Liu Y. Wallace G. G. (2010). Guidance of neurite outgrowth on aligned electrospun polypyrrole/poly(styrene-beta-isobutylene-beta-styrene) fiber platforms. *J. Biomed. Mater. Res. Part A* 94A 1004–1011. 10.1002/jbm.a.32675 20694967

[B19] Liu Z. M. Tang M. L. Zhao J. P. Chai R. J. Kang J. H. (2018). Looking into the future: toward advanced 3D biomaterials for stem-cell-based regenerative medicine. *Adv. Mater.* 30:e1705388. 10.1002/adma.201705388 29450919

[B20] Lu J. Zheng F. Y. Cheng Y. Ding H. B. Zhao Y. J. Gu Z. Z. (2014). Hybrid inverse opals for regulating cell adhesion and orientation. *Nanoscale* 6 10650–10656. 10.1039/c4nr02626h 25088946

[B21] McGinley L. M. Kashlan O. N. Bruno E. S. Chen K. S. Hayes J. M. Kashlan S. R. (2018). Human neural stem cell transplantation improves cognition in a murine model of Alzheimer’s disease. *Sci. Rep.* 8:14776. 10.1038/s41598-018-33017-6 PMC6170460 30283042

[B22] Mushtaq F. Torlakcik H. Vallmajo-Martin Q. Siringil E. C. Zhang J. H. Rohrig C. (2019). Magnetoelectric 3D scaffolds for enhanced bone cell proliferation. *Appl. Mater. Today* 16 290–300. 10.1016/j.apmt.2019.06.004

[B23] Musoke-Zawedde P. Shoichet M. S. (2006). Anisotropic three-dimensional peptide channels guide neurite outgrowth within a biodegradable hydrogel matrix. *Biomed. Mater.* 1 162–169. 10.1088/1748-6041/1/3/011 18458398

[B24] Omidinia-Anarkoli A. Boesveld S. Tuvshindorj U. Rose J. C. Haraszti T. De Laporte L. (2017). An injectable hybrid hydrogel with oriented short fibers induces unidirectional growth of functional nerve cells. *Small* 13:8. 10.1002/smll.201702207 28783255

[B25] Reidling J. C. Relano-Gines A. Holley S. M. Ochaba J. Moore C. Fury B. (2018). Human neural stem cell transplantation rescues functional deficits in R6/2 and q140 huntington’s disease mice. *Stem Cell Rep.* 10 58–72. 10.1016/j.stemcr.2017.11.005 29233555 PMC5768890

[B26] Rose J. C. Camara-Torres M. Rahimi K. Koehler J. Moeller M. De Laporte L. (2017). Nerve cells decide to orient inside an injectable hydrogel with minimal structural guidance. *Nano Lett.* 17 3782–3791. 10.1021/acs.nanolett.7b01123 28326790 PMC5537692

[B27] Severino F. P. U. Ban J. Song Q. Tang M. Bianconi G. Cheng G. S. (2016). The role of dimensionality in neuronal network dynamics. *Sci. Rep.* 6:29640. 10.1038/srep29640 PMC4939604 27404281

[B28] Shang Y. X. Chen Z. Y. Fu F. F. Sun L. Y. Shao C. M. Jin W. (2019). Cardiomyocyte-driven structural color actuation in anisotropic inverse opals. *Acs Nano* 13 796–802. 10.1021/acsnano.8b08230 30566827

[B29] Song C. G. Zhang Y. Z. Wu H. N. Cao X. L. Guo C. J. Li Y. Q. (2018). Stem cells: a promising candidate to treat neurological disorders. *Neural Regen. Res.* 13 1294–1304. 30028342 10.4103/1673-5374.235085 PMC6065243

[B30] Stachowiak A. N. Irvine D. J. (2008). Inverse opal hydrogel-collagen composite scaffolds as a supportive microenvironment for immune cell migration. *J. Biomed. Mater. Res. Part A* 85 815–828. 10.1002/jbm.a.31661 17937415

[B31] Tang M. L. Li J. He L. Guo R. R. Yan X. Q. Li D. (2019). Transcriptomic profiling of neural stem cell differentiation on graphene substrates. *Colloids Surfaces B Biointerfaces* 182:110324. 10.1016/j.colsurfb.2019.06.054 31288132

[B32] Xapelli S. Diogenes M. J. Crunelli V. Fitzsimons C. P. Vaz S. H. (2020). Editorial: glial and neural stem cells as new therapeutic targets for neurodegenerative disorders. *Front. Cell. Neurosci.* 14:71. 10.3389/fncel.2020.00071 32317936 PMC7147613

[B33] Xia L. Zhu W. J. Wang Y. F. He S. B. Chai R. J. (2019). Regulation of neural stem cell proliferation and differentiation by graphene-based biomaterials. *Neural Plast.* 2019:11. 10.1155/2019/3608386 PMC6817925 31737061

[B34] Xie J. W. Liu W. Y. MacEwan M. R. Bridgman P. C. Xia Y. N. (2014). Neurite outgrowth on electrospun nanofibers with uniaxial alignment: the effects of fiber density. Surface coating, and supporting substrate. *Acs Nano* 8 1878–1885. 10.1021/nn406363j 24444076 PMC4004310

[B35] Yang Y. R. Zhang Y. H. Chai R. J. Gu Z. Z. (2018). Designs of biomaterials and microenvironments for neuroengineering. *Neural Plast.* 2018:10. 10.1155/2018/1021969 PMC6304813 30627148

[B36] Zangi S. Hejazi I. Seyfi J. Hejazi E. Khonakdar H. A. Davachi S. M. (2016). Tuning cell adhesion on polymeric and nanocomposite surfaces: role of topography versus superhydrophobicity. *Mater Sci. Eng. C Mater. Biol. Appl.* 63 609–615. 10.1016/j.msec.2016.03.021 27040256

[B37] Zhang Y. Xia Y. N. (2012). Formation of embryoid bodies with controlled sizes and maintained pluripotency in three-dimensional inverse opal scaffolds. *Adv. Funct. Mat.* 22 121–129. 10.1002/adfm.201101690

[B38] Zhang Y. S. Regan K. P. Xia Y. N. (2013). Controlling the pore sizes and related properties of inverse opal scaffolds for tissue engineering applications. *Macromol. Rapid Commun.* 34 485–491. 10.1002/marc.201200740 23365045

[B39] Zhang Y. S. Zhu C. L. Xia Y. N. (2017). Inverse opal scaffolds and their biomedical applications. *Adv. Mater.* 29:10.1002/adma.201701115. 10.1002/adma.201701115 PMC5581229 28649794

[B40] Zhu S. M. Lu T. Zhang D. (2017). Progress in stimuli-responsive photonic crystals with biological structures. *Acta Polym. Sin.* 2017 229–244.

[B41] Zuidema J. M. Kumeria T. Kim D. Kang J. Y. Wang J. A. N. Hollett G. (2018). Oriented nanofibrous polymer scaffolds containing protein-loaded porous silicon generated by spray nebulization. *Adv. Mat.* 30:e1706785. 10.1002/adma.201706785 PMC6475500 29363828

