# Supplementary material for: Oriented Neural Spheroid Formation and Differentiation of Neural Stem Cells Guided by Anisotropic Inverse Opals
Source: Front Bioeng Biotechnol. 2020 Jul 31;8:848. doi: 10.3389/fbioe.2020.00848 (PMC7411081; doi:10.3389/fbioe.2020.00848)
Supplement: Supplementary file 1 [file Image_1.pdf]

Supporting Information

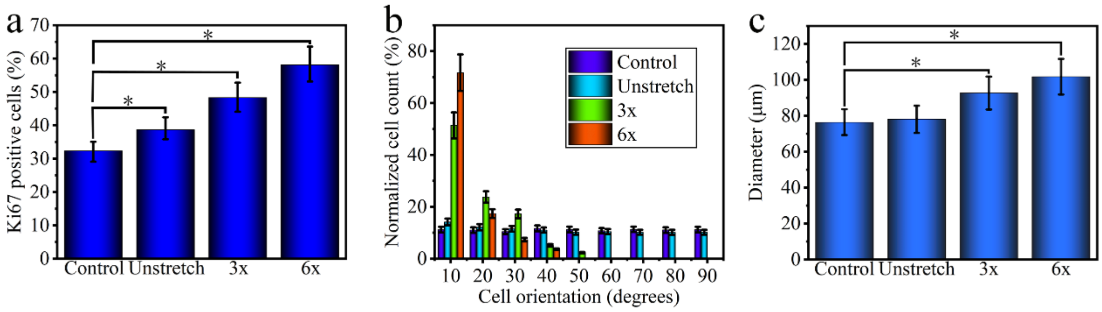

Figure S1. The percentage of Ki67-positive neural stem cells (**a**), the orientation angles of the neural stem cells (**b**), the average diameter of the neural spheroids **(c)**.

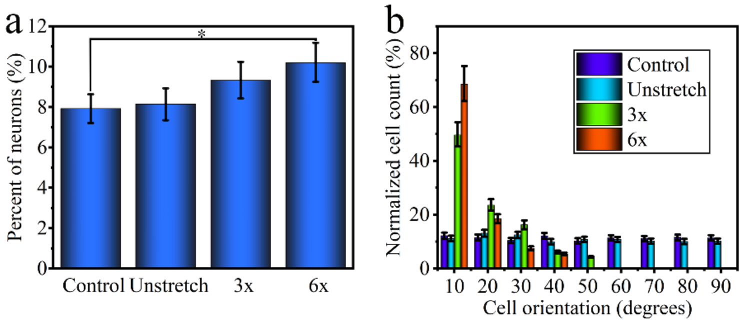

Figure S2. The percentage of newborn neurons among all the differentiated cells (**a**) and the orientation angles of the newborn neurons (**b**).

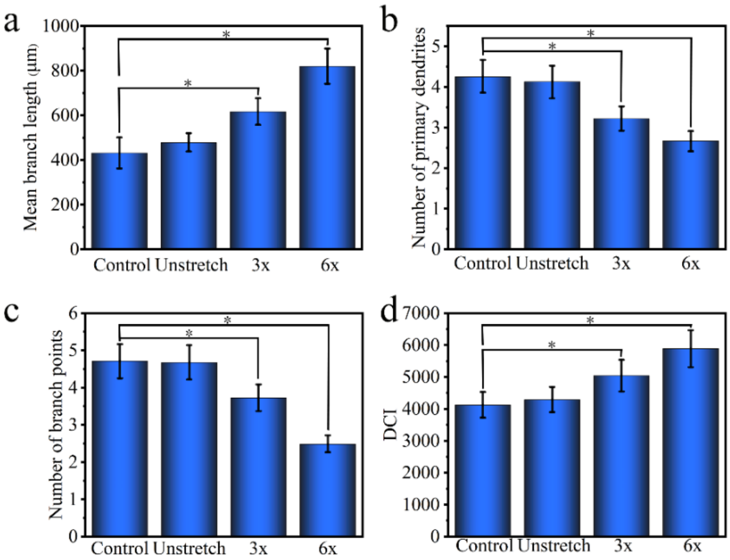

Figure S3. Morphological properties of the newborn neurons. **a:** The average branch length of the

newborn neurons. **b:** The average number of primary dendrites of the newborn neurons. **c:** The average number of branch points of the newborn neurons. **d:** The DCI of the newborn neurons. (DCI, formula:(∑branch tip orders+number of branch tips)/(number of primary dendrites)×(total arbor length)).
